# Visualized Computational Predictions of Transcriptional Effects by Intronic Endogenous Retroviruses

**DOI:** 10.1371/journal.pone.0071971

**Published:** 2013-08-06

**Authors:** Ying Zhang, Artem Babaian, Liane Gagnier, Dixie L. Mager

**Affiliations:** 1 Terry Fox Laboratory, British Columbia Cancer Agency, Vancouver, British Columbia, Canada; 2 Department of Medical Genetics, University of British Columbia, Vancouver, British Columbia, Canada; Louisiana State University, United States of America

## Abstract

When endogenous retroviruses (ERVs) or other transposable elements (TEs) insert into an intron, the consequence on gene transcription can range from negligible to a complete ablation of normal transcripts. With the advance of sequencing technology, more and more insertionally polymorphic or private TE insertions are being identified in humans and mice, of which some could have a significant impact on host gene expression. Nevertheless, an efficient and low cost approach to prioritize their potential effect on gene transcription has been lacking. By building a computational model based on artificial neural networks (ANN), we demonstrate the feasibility of using machine-learning approaches to predict the likelihood that intronic ERV insertions will have major effects on gene transcription, focusing on the two ERV families, namely Intracisternal A-type Particle (IAP) and Early Transposon (ETn)/MusD elements, which are responsible for the majority of ERV-induced mutations in mice. We trained the ANN model using properties associated with these ERVs known to cause germ-line mutations (positive cases) and properties associated with likely neutral ERVs of the same families (negative cases), and derived a set of prediction plots that can visualize the likelihood of affecting gene transcription by ERV insertions. Our results show a highly reliable prediction power of our model, and offer a potential approach to computationally screen for other types of TE insertions that may affect gene transcription or even cause disease.

## Introduction

ERV-related sequences and other sequences derived from TEs comprise large fractions of the human and mouse genomes [1], [2]. While new TE insertions can obviously physically disrupt coding exons and destroy gene function, their effects within introns are extremely variable. For example, over 40 published cases of mouse germ line mutations are due to effects of new ERV insertions within introns, resulting in abnormal transcript processing and a measurable phenotype [3]. On the other hand, the high prevalence of very similar ERVs fixed within introns suggests that, frequently, a gene can tolerate such intronic insertions without significant detrimental effects. Although aberrant gene transcription caused by *de novo* TE insertions has been reported for various TE types/families, most examples documented in the literature are from ERVs in mice, probably due to their high activity in the host genome [3] and the wide use of mouse inbred strains as disease models. Considering the availability of data based on known mutagenic TE insertions, we focused on ERVs in mice in our study, but the same strategy may be extended to other TEs in more species including humans.

Several recent efforts have catalogued thousands of ERVs that are variably present (i.e. insertionally polymorphic) in different mouse strains [4]–[6]. Since some of these polymorphic ERVs have been shown to contribute to strain-specific traits [7], [8], it would be useful to develop computational methods to predict which are the most likely to impact host genes. To this end, we have designed a computational model based on artificial neural networks (ANN) and generated a set of highly reliable prediction plots that can directly evaluate the likelihood of affecting gene transcription by intronic ERV insertions.

## Results and Discussion

### Rationale

The potential of intronic TEs to affect gene transcription is likely determined by multiple factors. For example, the orientation of ERVs relative to host genes is related to the probability of disrupting normal transcription [9]–[12]. Generally, most ERVs fixed in both human and mouse genomes show an antisense bias in gene introns, likely due to their less harmful effects on gene transcription and, therefore, a greater probability to escape removal by negative selection. In agreement with this observation, a majority of ERVs causing mutations or disease are indeed in the sense orientation with respect to the enclosing gene [11], [13], supporting the view that orientation of ERVs is an important factor influencing their potential effects on gene transcription. Another factor is the location of the element within an intron. Our previous study of TE distributions in gene introns found an underrepresentation of TEs near intron-exon boundaries, suggesting greater negative selection against insertions in such regions [13]. Other potential factors that could affect the probability of a TE disrupting gene transcription include TE size, type of the nearby splice site (i.e. splice donor or acceptor), number and strength of cryptic splice sites within the TEs [13]. While it has been shown that the function and expression pattern of target genes may also contribute to the effects of TE insertions [14]–[17], in this study we focus only on factors related to the properties of TE insertions.

Given the complexity of influential factors, we constructed a computational model to predict which ERV insertions have the highest potential to affect gene transcription. As one of the most powerful machine-learning methods for solving complex classification problems, an artificial neural network (ANN) has many advantages including its ability to model high-dimensional non-linear data space, take both numeric and categorical inputs, work in highly flexible model structures, and produce reliable outputs with a reasonable cost of training [18]. In this report, we show the feasibility of training an ANN model that can evaluate the possibility of a given ERV affecting gene transcription in mouse, as well as the biological interpretation of the output space and some of its future potential applications.

### Selection of positive and negative datasets for training

As for any mathematical classifiers based on supervised-training techniques, generating clean, well-defined training datasets is the first, and arguably the most critical, step in building a successful model. Here, we have carefully collected mouse mutagenic ERV insertions in gene introns that have been reported in the literature to cause a phenotype, and selected 33 of them as the positive dataset after removing cases with incomplete or inaccurate information (Table S1). In addition, all positive ERV insertions chosen here are from either the IAP or the ETn/MusD family, both of which belong to Class II ERVs [19] and in total account for more than 80% of the germ line mutagenic ERV insertions reported in the literature (estimation based on Table S3 of [13]). To further simplify our study, we only included in our positive dataset intronic ERV insertions causing transcriptional disruption (e.g. alternative splicing or premature polyadenylation) of the enclosing gene. All of these mutagenic ERVs are recent *de novo* insertions identified only in individual mice, and are full-length copies carrying the intact ERV sequence (5–8 kb).

As our negative training set, we chose intronic ERV loci present in at least four mouse strains [4], assuming that such ERVs are less likely to cause significant transcriptional effects. Since one cannot rule out the potential influence some common ERVs could have on nearby genes, we have depleted our dataset of any ERVs showing EST evidence of chimeric transcripts between the ERV and the host gene, which further ensures their neutral effect on genes. To be comparable to the positive training data, only near full-length (>5 kb) elements were selected. In total, we collected 117 such ERV insertions in the mouse reference genome as negative cases for ANN training (Table S2).

### Selection of input factors for ANN training

**Figure 1:**
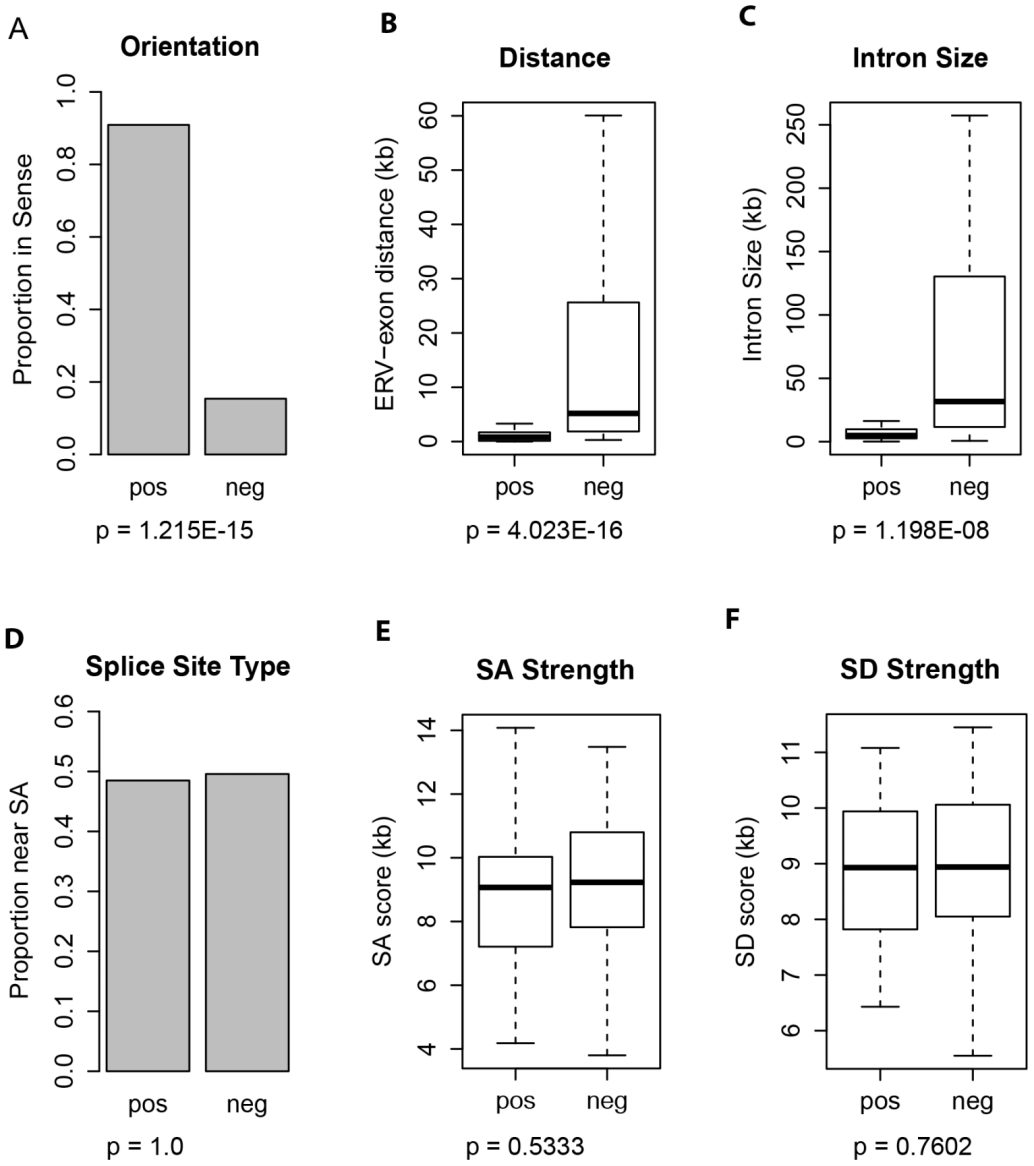
Comparisons of potential factors linked to the likelihood of affecting gene transcription by ERVs. The name of factors is given above each panel, and the average value of each factor is compared between the positive and negative datasets. Panel A and D are comparisons of proportions using bar plots, and p-values are calculated using the ‘equality of proportions test’; Panel B, C, E and F are comparisons of means using box plots, and p-values are calculated using the ‘Student's *t*-test’.

In order to build a neural network model that can be trained effectively and make reliable predictions selection of relevant input factors is also important. To achieve this, we first compared a panel of potential factors between the positive and negative datasets, and found statistically significant differences for the orientation of ERV, distance from ERV to exon, and intron size (Figure 1A, B & C). However, we did not detect a significant difference for the type of the nearest splice site (i.e. splice donor or acceptor) to ERVs between the two data classes (Figure 1D), possibly because this factor is only relevant when TEs are in very close proximity (∼20 bp) to intron/exon boundaries [13]. Since we hypothesized that introns with stronger splice site signals may show a higher tolerance for intronic ERV insertions, we also tested the strength of splice signals at intron/exon boundaries using MaxEntScan [20], but found no difference between positive and negative cases (Figure 1E & F). Due to a lack of information for the ERV DNA sequence itself for most of the positive training data, we could not consider any sequence-based features associated with the ERVs except the type/family of ERVs in general (i.e. ETn/MusD or IAP). In fact, when we compared the training results between using only the three factors showing significant difference between positive and negative datasets versus using all the above factors, there was a slight decrease in both ANN performance and training efficiency for the latter (data not shown). Based on the above analysis, we decided to use a total of four factors, namely the orientation, distance to exon, intron size, as well as the ERV family, as the input factors for ANN training.

### Construction and Training of the ANN model

To reliably predict which intronic ERV insertions may affect gene transcription, a computational classifier that can correctly differentiate positive and negative datasets based on supervised learning is required. Here we used an ANN architecture known as multiple layer perceptron (MLP; see Figure S1), which is widely used for solving classification problems [21]. As described in the Materials and Methods, our MLP model can take the four selected factors of a given ERV insertion as its four inputs, and produce a predicted likelihood of causing transcriptional disruption by that ERV insertion.

However, as with many other machine-learning techniques, two major innate technical problems exist for MLP training. The first problem is the possible ‘over-fitting’ of training results based on limited data. In this scenario, the trained neural network may perform extremely well based on training data, but show relative poor performance for data unseen before. Here we applied a 3-fold cross-validation (see Methods), which provides three sets of partially overlapped training data, as well as three datasets reserved for testing. In this way, the performance of each MLP model can be evaluated objectively with unseen data. Another potential problem is the local maximum effect. Since the process of assigning initial parameters (i.e. connection weights between neurons) for the neural network is random, the performance of the prediction model after training may vary significantly. To handle this problem, we applied randomization at two different levels during ANN training. First, we randomly permutated both the positive and negative data before creating subgroups for the 3-fold cross-validation. Such process was repeated for 10 times, resulting a total of 30 datasets for ANN training and testing. In addition, we also applied randomization of initial parameters of the MLP model 100 times, increasing the total number of ANNs for training up to 3000 (see Figure S2 for the conceptual workflow). After training, the model can be used for performance evaluation or making predictions based on unseen data by taking an average output of these randomized ANNs. Using receiver operating characteristic (ROC) curve analysis based on the testing datasets, we showed an overall ‘area under the curve’ (AUC) larger than 0.99 (Materials and Methods), indicating that our well-trained MLP model has a high capability of correctly discriminating the unseen positive ERV data from negative cases.

### Performance evaluation and threshold optimization

**Figure 2:**
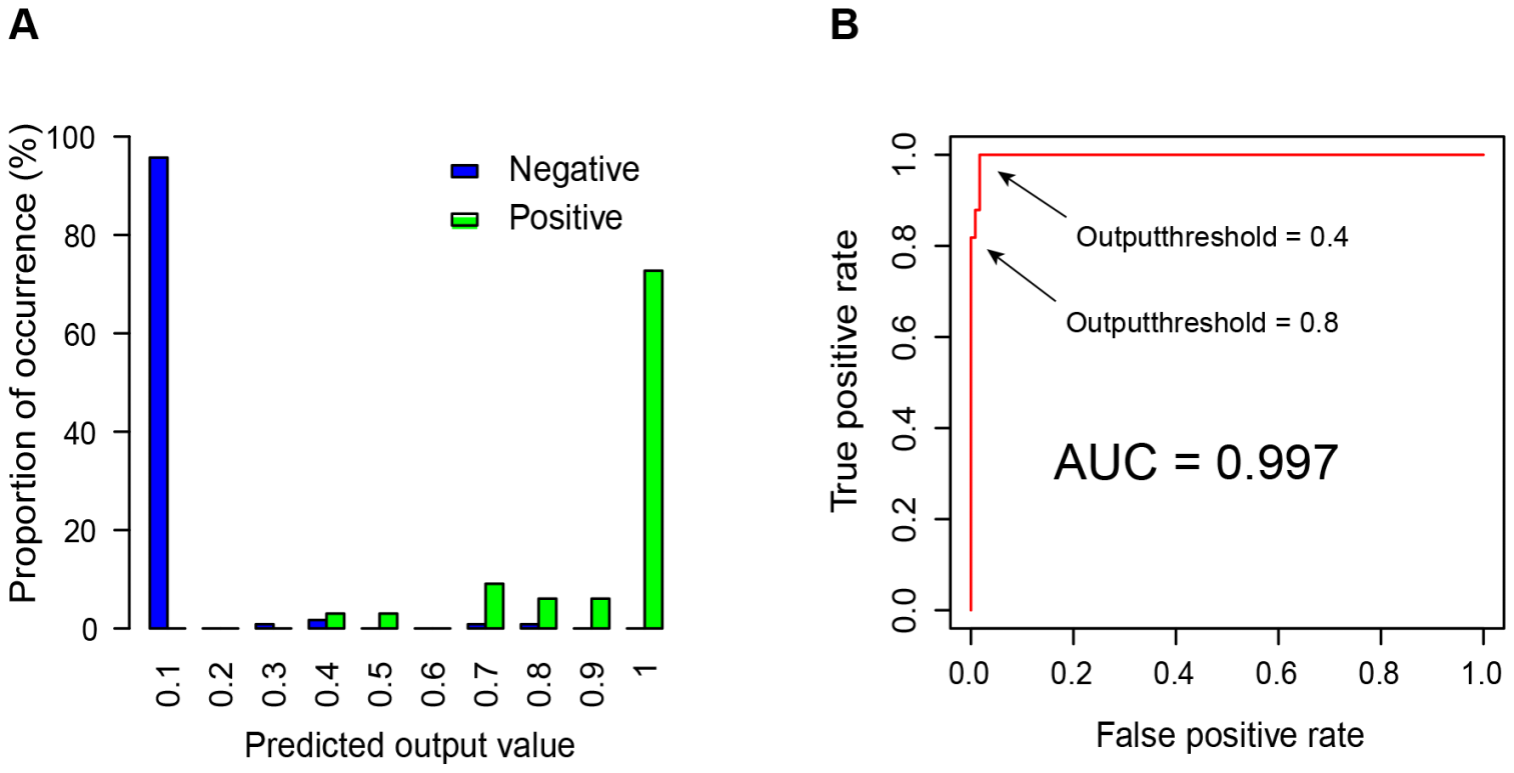
Selection of the cutoff threshold for discriminating positives and negatives. A) the distribution of predicted outcomes of the positive and negative datasets. Each bar corresponds to a given range of predicted likelihood of affecting transcription by ERVs, and the height of the bar represents the percentage of ERV insertions with a predicted value within that range. Green and blue bars represent positive and negative data, respectively. Panel B shows the performance of the trained MLP model using ROC curve analysis based on the positive and negative datasets. AUC stands for ‘the area under the curve’. As shown by the two arrows in the figure, when the cutoff threshold is set at 0.4, the model's true positive rate reaches 1; when the cutoff threshold is set at 0.8, the model's false positive rate is 0.

To further examine the relationship between the accuracy of prediction and selection of output threshold, we calculated the averaged outputs of our trained MLP model using all the 150 training cases (33 known positive and 117 negative), and plotted the distribution of the predicted values (Figure 2A). Since the output target of our training data is either ‘1’ for positive or ‘0’ for negative, ‘0.5’ is likely a reasonable cutoff threshold to discriminate between positives and negatives. Indeed, by taking ‘0.5’ as the classification boundary, our MLP model gives a false positive rate (FPR) as low as 1.7% and, at the same time, maintains a true positive rate (TPR) as high as 96%. To examine the overall prediction power of our MLP model at a more general level, we plotted the ROC curve based on the same dataset and obtained an AUC value larger than 0.997 (Figure 2B). According to the ROC curve, when the output threshold is set to ‘0.4’, the maximum sensitivity can be reached (TPR = 100%) while the model still maintains a specificity (i.e. 1-FPR) of 98.3%; on the other hand, when the cutoff threshold is set to ‘0.8’, the model shows its maximum specificity (FPR = 0%) along with a sensitivity of 81.8%. Although the data used for performance evaluation here are the same as used for ANN training, a major ‘over-fitting’ effect is unlikely since the model has already been cross-validated. Nonetheless, the above result not only shows the high performance of our MLP model, but also demonstrates the tradeoff between sensitivity and specificity when choosing the “optimized” cutoff threshold.

### Predicting the transcriptional effects of polymorphic ERV insertions

Having our MLP model well trained, it would be interesting to computationally predict the possibility of affecting gene transcription by unknown ERV insertions. Since insertionally polymorphic ERVs are not present in all mouse strains, they are generally younger than fixed elements and are more likely to impart functional effects that are still under selection [4]. For the polymorphic dataset, we included all full-length intronic ERV insertions that are present in the C57BL/6J but absent from at least one of three other mouse strains; A/J, DBA/2J, and 129X1/SvJ [4]. In total, we obtained 134 cases (Table S3), with eight ETn/MusD elements and the rest belonging to the IAP family.

Running our trained MLP model on the 134 polymorphic ERV insertions, we predicted if each insertion will affect gene transcription (Table S3). Using the ‘0.5’ cutoff threshold, 11 out of 134 (8.2%) polymorphic ERV insertions were predicted to be ‘positive’, indicating a higher likelihood of influencing transcription of the enclosing gene. Remarkably, when we manually reviewed each of the 134 polymorphic ERV insertions in the literature (see Materials and Methods), we found that 4 of the 11 cases (36%) predicted as ‘positive’ have been confirmed as significantly affecting the transcription of gene (see references attached to Table S3). In comparison, among the remaining 123 polymorphic ERV insertions, only 5 (4%) have been reported as disrupting gene transcription (p = 0.0005, proportion equality test). This result clearly indicates the significance of our approach in predicting positive ERV insertions from unknown data.

**Figure 3:**
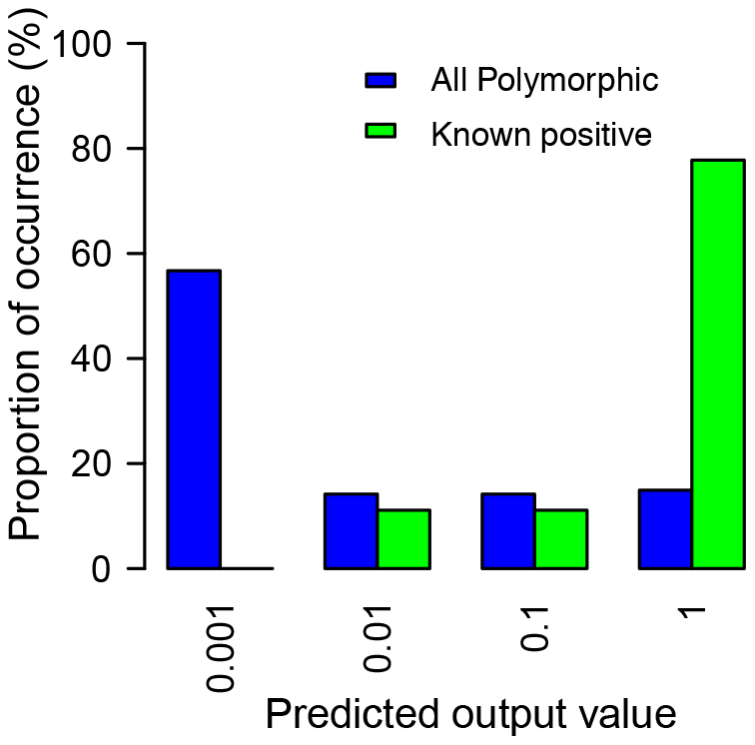
Distribution of the predicted likelihood of affecting gene transcription by polymorphic ERV insertions. Each bar corresponds to a given range of predicted likelihood of affecting transcription by ERVs, and the height of the bar represents the percentage of ERV insertions with a predicted value within that range. Blue bars represent the distribution of all 134 polymorphic ERV insertions chosen for this study. Green bars represent the distribution of only polymorphic ERV insertions known in the literature as disrupting gene transcription. The ranges of predicted values (horizontal axis) are based on the logarithm scale to increase resolution at the low-end of predicted values.

Noticeably, the distribution of the predicted outputs of the 134 polymorphic ERV insertions (blue bars in Figure 3) is highly skewed toward very small values (e.g. more than half of these cases have a predicted output value less than 0.001). This observation is in agreement with other data showing that, in general, insertionally polymorphic ERVs show the signatures of purifying selection [5], albeit a small number of slightly deleterious elements may be tolerated in the experimental mouse populations. On the contrary, seven of the nine positive polymorphic ERV insertions confirmed in the literature (green bars in Figure 3) have a predicted output larger than 0.1, and none is smaller than 0.001. While this apparent difference between the two distributions clearly shows the prediction power of our MLP model on unseen datasets, it also indicates that the optimized cutoff threshold may be adjusted to a value much lower than 0.5 to gain the most of sensitivity without losing much specificity.

### In silico ERV insertion simulations reveal biological interpretation of the ANN model

**Figure 4:**
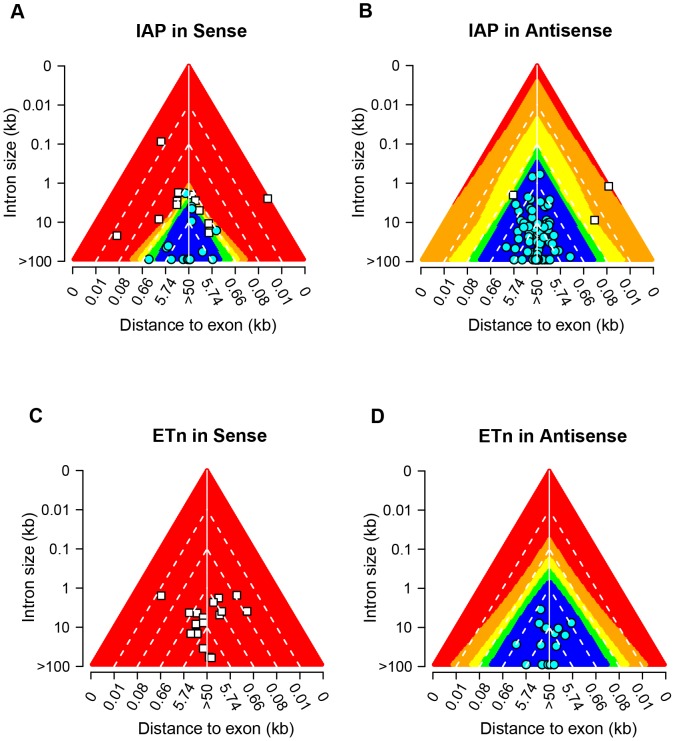
Output space analysis of MLP predictions. The theoretical output space of the MLP prediction model is plotted separately using *in silico* ERV insertions for A) IAP in sense, B) IAP in antisense, C) ETn in sense, and D) ETn in antisense. Different ranges of predicted output values are illustrated by a rainbow of colors as shown at the bottom of the figure. For each of the above figure panels, the main triangle plot can be viewed as a stack of ERV-containing introns aligned by their centers, with the horizontal axis showing the distance from a given ERV insertion to its nearest exon. The Vertical axis represents the size of intron, with large introns at the bottom and small ones on top. All known positive (filled circles in cyan) and negative (filled squares in white) cases used for ANN training are also superimposed on top of each plot according to the type and orientation of ERVs.

Although our ANN performance analysis and computational predictions based on polymorphic ERV data showed a reliable classification capability of the MLP model, the effects and interactions of input factors are only implied by a set of numeric connection weights between artificial neurons (i.e. the ‘black box’ phenomenon of ANN), making it very difficult to derive biological interpretations of the results (Figure S1) [18]. To cope with this problem, we designed a computational experiment using artificial ERV insertions covering the entire 4-dimensional searching space of input factors (see Materials and Methods) which revealed interesting output patterns. As shown in Figure 4, *in silico* insertions of different ERV families and orientations were examined separately, with each subgroup only considering the input factors of intron size and distance of the ERV insertion to the nearest exon. For example, Figure 4A & B shows the output space of sense- and antisense-oriented IAP insertions, respectively, with different ranges of output represented by different colors. When the two plots (4A vs. 4B) are compared side-by-side, the most striking difference is the ratio between the red/orange (likely positive) and green/blue (likely negative) areas, indicating a much higher likelihood of imposing transcriptional effects for sense-oriented IAPs. Since most of the ready-to-use transcriptional signals within an ERV element are in the sense orientation, its confirmation with our ANN model indicates the feasibility of using these simulations of *in-silico* ERV insertions to infer biological consequences.

Furthermore, when the factors of intron size and distance to the nearest exon are examined independently for both sense and antisense IAP insertions, a clear trend of smaller intron size and shorter ERV-exon distance can be observed for positive ERV insertions regardless of the orientation (Figure 4A & B). For example, when an IAP is inserted in an intron smaller than 50 bp, it is highly likely to have an effect on gene transcription no matter if it is inserted in sense or antisense. However, when the intron is large (e.g. >100 kb), an antisense IAP is very likely to be a negative/neutral insertion unless it is extremely close to the intron-exon boundary. One potential explanation for such anobservation is the apparent higher possibility of TE insertions in large introns being far away from intron/exon boundaries, but other hypothetical factors such as a higher tolerance of large introns to cryptic splice sites may also be involved. Moreover, while the same pattern exists for sense IAPs, the intron size effect is much smaller compared to the antisense insertions. Similarly, if one looks at the factor of ERV-exon distance, a clear trend of a higher opportunity to interrupt gene transcription can be observed for an intronic IAP insertion near exons, albeit such distance can be extended much further for sense-oriented insertions (Figure 4A). The same analysis for the ETn/MusD family basically showed similar observations, except an overall higher chance of disrupting gene transcription compared to IAPs with similar input factors (Figure 4C & D). Strikingly, our computational simulation of sense-oriented ETn/MusD insertions revealed an ‘all-positive’ output space, which suggests that sense insertion of an ETn/MusD element is very likely to disrupt the normal transcription of it's enclosing gene (Figure 4C).

### Using prediction plots to visually evaluate the transcriptional effects of ERVs

As shown in Figure 4, the ANN prediction plots derived from our *in silico* ERV insertion simulations cover the entire ANN input-output space. To evaluate the feasibility of using these prediction plots to visually estimate the likelihood of affecting gene transcription by given ERV insertions, we plotted all the known ERV insertions used for ANN training on top of the output space of our computational simulations. As expected, for both IAP and ETn/MusD insertions in either sense- or anti-sense orientation, the predicted output zone of these elements largely agrees with their real data class (Figure 4). Notably, all sense-oriented ETns in our training data are indeed positive insertions, which were all within the strong positive output region (red area in Figure 4D) as predicted. On the other hand, all antisense-oriented ETns are negative insertions, with all located within the strong negative regions (blue area in Figure 4C). In fact, among all the known positive ERV insertions collected here, three are located more than 10 kb away from the nearest exon/intron boundary but can still disrupt gene transcription, and they are all sense-oriented elements from the ETn/MusD family, showing a strong, dominate orientation effect for this ERV type. Indeed, when we calculated the proportion of full-length ERVs in genes in the C57BL/6 reference genome, we found only 7.7% (3 of 39) of intronic ETn/MusD elements are in sense orientation compared to 21.5% (53 of 247) of IAPs in sense, indicating a much higher antisense-orientation bias for the genomic distribution of ETns/MusDs in genes.

Since our model predicts that full-length ETn/MusD elements in the sense orientation are highly likely to disrupt transcription, we more closely examined the 3 such cases detected in the reference genome to look for unusual features. One of these cases is within an “inferred” Refseq gene, *Vmn2r100*, that has no corresponding mRNAs or ESTs, calling the validity of the gene into question. The second case, within the *Sgk1* gene, was called as a “full length” ETn by our computational screen but is actually two solitary LTRs separated by several kb. Moreover, approximately half of the ESTs for this gene actually terminate at the polyA signal within one of these LTRs, suggesting significant transcriptional effects of this LTR. The third ETn case, in the *Sult2a2* gene, is within a large 40 kb intron that is ∼85% repetitive, suggesting that this intron is relatively resistant to TE-mediated transcriptional disruptions.

### Concluding remarks

The transcriptional effects imparted by ERVs and other types of transposable elements are widely appreciated, and the mechanisms of TE-induced transcriptional interference are becoming better understood [3], [22]. As more insertionally polymorphic/private TE insertions are identified, it would be useful to computationally generate a priority list of TEs affecting gene transcription for further biological examinations. In addition, with the accumulation of evidence that TEs may also play beneficial roles in gene transcription within normal cells (i.e. TE exaptation) [23], it would also be interesting to computationally identify new examples of co-opted TEs on a genome-wide scale. In this study, we developed a computational model based on artificial neural networks that can predict the likelihood of affecting gene transcription by ERV insertions in mice, and provided a set of prediction plots that can be easily used for visually evaluating the potential effects of intronic ERVs.

Importantly, the same strategy could be adopted for other TEs and species when data become available. For example, recent efforts to catalog polymorphic/private TE insertions in the human genome by different research groups have generated lists of thousands of polymorphic LINEs (long interspersed nucleotide elements) and SINEs (short interspersed nucleotide elements) in humans [24]–[27], and it would be of value to predict the probability that a given element is associated with disease susceptibility or phenotypic variability. Moreover, somatic TE insertions in cancer [28] or tissues such as brain [29], [30] are also being documented. To apply our method to human TEs, sufficient numbers of well-studied mutations/diseases caused by intronic TE insertions are required to use as positive training data. A recent review [22] lists 13 cases of new intronic insertions of Alu SINEs that cause disease but only six L1 LINE intronic cases, which is not yet sufficient for training a model. Bearing in mind that any transcriptional effects of polymorphic intronic TEs may be subtle, complimentary methods to identify such “transcription-altering” or “disease-associated” TEs might include individual-specific whole transcriptome analysis to screen for TE-gene chimeric transcripts or disease association studies. By applying a combination of approaches, it should be possible to identify polymorphic or somatic TEs most likely to have a functional impact.

## Materials and Methods

### Neural network construction

We applied a 3-layer MLP neural network to discriminate the positive ERV insertions from negative ones. As illustrated in Figure S1, the first layer is an input layer with four nodes (neurons), which can take values of the four different input factors respectively; the middle layer is a hidden layer of three neurons mutually connected to all the other neurons in both the input and output layers; the last layer is the output layer, which consists of only a single neuron as the output node. As in most MLPs, it uses the back-propagation algorithm to adjust connection weights between neurons so that the output error is minimized. Here we used the R package ‘*neuralnet*’ [31] to build the model.

### Data preprocessing

To improve the training quality, all training and testing data were standardized before taken by the ANN. For orientation, we used ‘1’ for sense-oriented ERVs and ‘0’ for antisense ones. Similarly, ERVs belonging to the ETn/MusD family are defined as ‘1’, and ‘0’ for those from the IAP family. Since we reasoned that the biological relevance of intron size would be unlikely to show a significant difference between introns ≥100 kb, any introns larger than this size were considered as 100 kb. Then, we used log_10_ for the length of each intron to correct the non-normal genomic distribution of intron size, and divided it by log_10_(100 kb) so that introns ≥100 kb are all normalized to ‘1’. In this way, any intron between 0–100 kb was normalized to a value between [0, 1]. The same data transformation was applied to the ‘ERV-exon distance’ with the maximum distance set as 50 kb, which is half of the maximum intron size. Finally, the target output of ANN was also defined as a Boolean value of ‘1’ or ‘0’, which represents the data class of ‘positive’ and ‘negative’, respectively.

### Neural network training with cross validation

In order to properly train and evaluate the MLP neural network, we applied a 3-fold cross validation during the ANN training. First, all the 33 positive and 117 negative cases were divided into three subgroups with an equal group size, respectively (i.e. 11 cases/subgroup for positive cases, and 39 cases/subgroup for negative cases). Then, one subgroup of positive and one subgroup of negative cases were selected and mixed to build the test dataset, which in total consists of 50 cases and was denoted as Testing Dataset A. The remaining two subgroups of positive cases and two subgroups of negative cases were mixed and denoted as the Training Dataset A, which consists of 100 cases in total. During ANN training, only the 100 cases of training dataset (i.e. Training Dataset A) were presented to the MLP model, while the 50 cases of testing dataset (Testing Dataset A) were reserved for post-training performance evaluation. Finally, the same process was repeated two more times, with each time selecting a different subgroup as the testing dataset (Training dataset B & C; Testing Dataset B & C).

### Data consolidation and model performance evaluation

After randomization, as many as 30 MLP models were trained based on different combinations of positive and negative data. In addition, each of the 30 MLP models was reinitialized and retrained 100 times using the same training dataset to avoid potential initialization bias. Since the output of each MLP is a continuous number between [0, 1] (which reflects the likelihood of the input ERV affecting gene transcription), a consolidated result can be calculated by taking the average of multiple MLP outputs. To evaluate the performance of our MLP model, we used the receiver operating characteristic (ROC) package in R (ROCR) and calculated the average value of the area under the curve (AUC) using the reserved test datasets from the 3-fold cross-validation. To simplify the calculation, we first consolidated outputs from the 100 reinitialized MLPs as mentioned above, and then performed ROC analysis for each of the 30 MLP models trained with unique datasets from cross-validation. As a result, we observed an averaged AUC value of 0.990 with a standard deviation of 0.011.

### In Silico ERV insertion simulation

To evaluate the biological relationships between input factors and the ANN prediction, we generated artificial ERV insertion data by covering the entire 4-dimentional input space of our MLP model. Specifically, for each combination of a given ERV type and orientation, we equally divided the after-normalization input range of [0, 1] into 100 units for each of the two continuous input factors (i.e. intron size and ERV-exon distance), generating a 100×100 grid of input data points. Then, each data point was presented to the trained MLP model, and an averaged output prediction was calculated. Taking into account the four combinations of the other two Boolean input factors (i.e. ERV type and orientation), a total of 40,000 artificial ERV insertions and their corresponding predictions were generated.

### Literature searches on polymorphic ERVs

To look for published evidence that any of our set of 134 polymorphic ERVs in introns affect gene transcription, PubMed searches were conducted for each gene using keywords including the gene name, “insertion”, “IAP”, “ETn”, “ERV” and “transposon”. In order to conduct a more thorough search of other resources such as preprint archives, conference proceedings, books, databases and supplementary material the same keywords were used to search Google and Google Scholar. We did not count cases as “positive” if the only evidence was appearance on a list of ERV-gene chimeric transcripts (for example) without experiments to quantify the transcriptional effect.

### Source code of software

Instead of trying to develop a mature software package for biologists to use, the original intention of this study was to investigate the methodological possibility of making predictions of the potential transcriptional effects of TEs using computational approaches. However, in order to help other researchers to validate or extend our work, all software source codes for ANN training and prediction can be provided upon request.

## Supporting Information

Figure S1 **The architecture of the Multi-Layer Perceptron (MLP) model.**
(TIF)

Figure S2 **The conceptual workflow of ANN training and prediction of ERVs affecting transcription.**
(TIF)

Table S1 **Mouse mutagenic ERV insertions used as positive data for ANN training.**
(DOCX)

Table S2 **Mouse common ERV insertions used as negative data for ANN training.**
(DOCX)

Table S3 **Mouse polymorphic ERV insertions with predicted likelihood of affecting gene transcription.**
(DOCX)
