# Supplementary figures and images for: Visualized Computational Predictions of Transcriptional Effects by Intronic Endogenous Retroviruses
Source: PLoS One. 2013 Aug 6;8(8):e71971. doi: 10.1371/journal.pone.0071971 (PMC3735543; doi:10.1371/journal.pone.0071971)

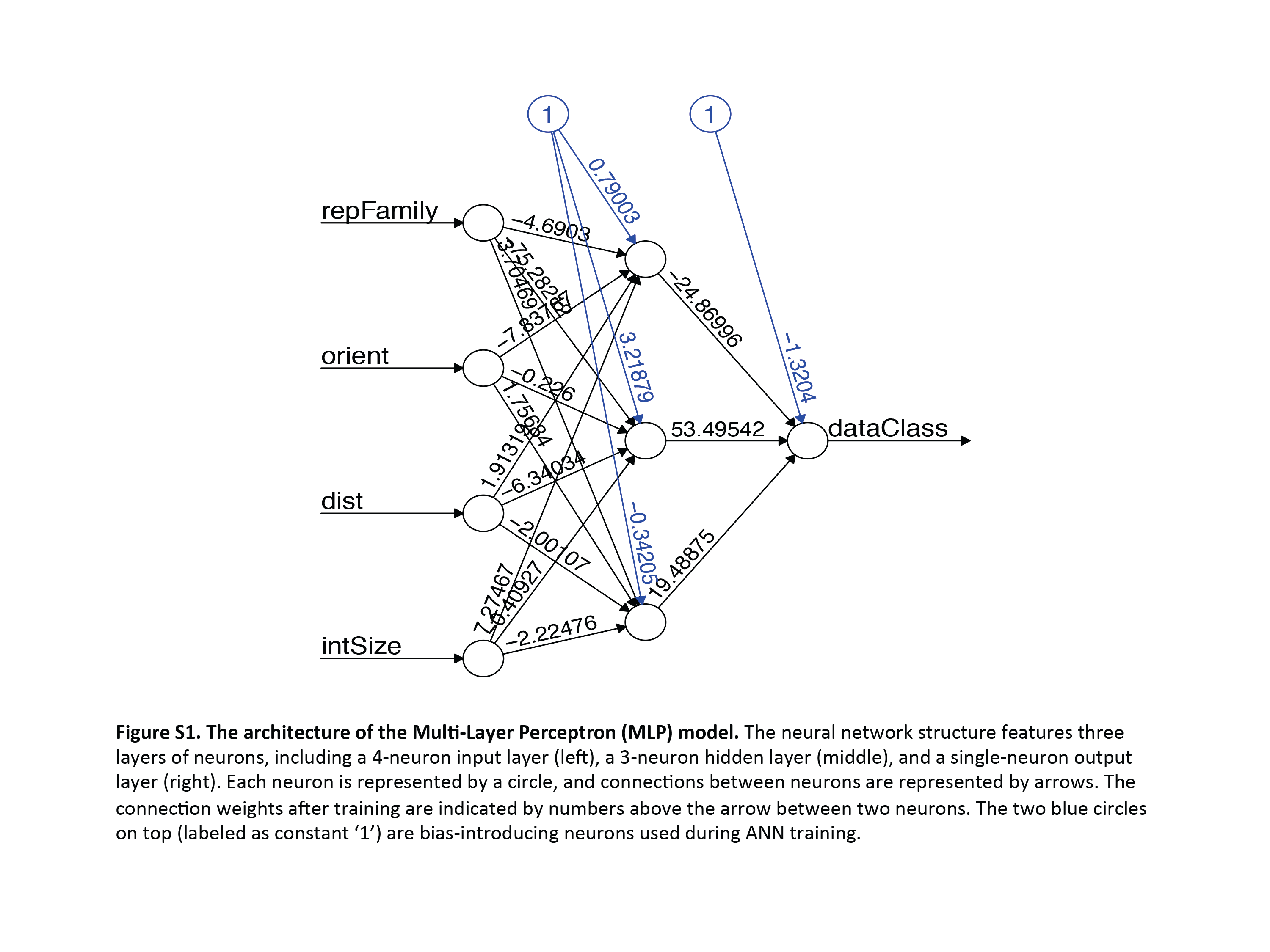

Supplement: Figure S1 — The architecture of the Multi-Layer Perceptron (MLP) model. (TIF) [file pone.0071971.s001.tif]

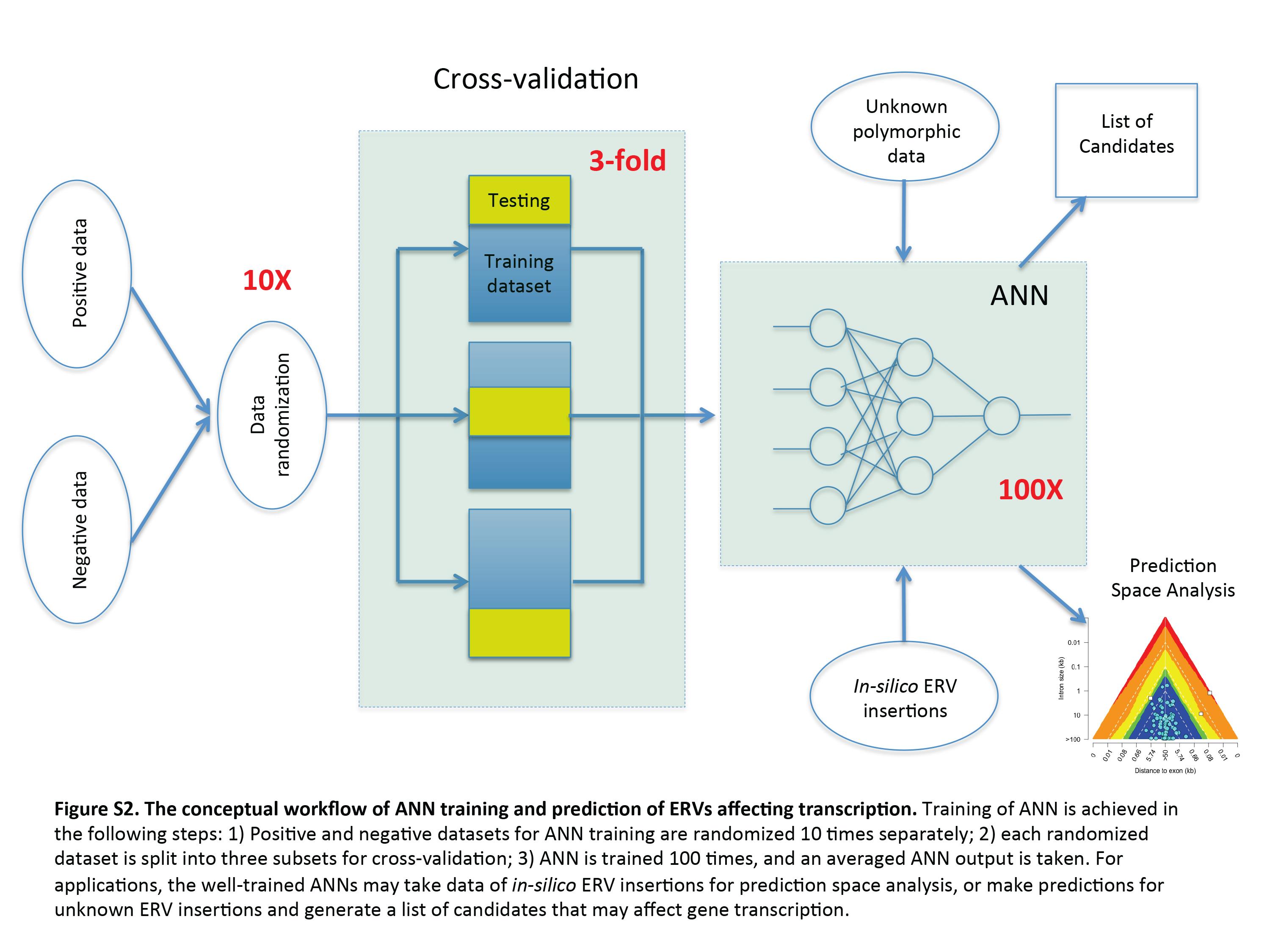

Supplement: Figure S2 — The conceptual workflow of ANN training and prediction of ERVs affecting transcription. (TIF) [file pone.0071971.s002.tif]
