# Supplementary material for: Visualized Computational Predictions of Transcriptional Effects by Intronic Endogenous Retroviruses
Source: PLoS One. 2013 Aug 6;8(8):e71971. doi: 10.1371/journal.pone.0071971 (PMC3735543; doi:10.1371/journal.pone.0071971)
Supplement: Table S1 — Mouse mutagenic ERV insertions used as positive data for ANN training. (DOCX) [file pone.0071971.s003.docx]

**Table S1. Mouse mutagenic ERV insertions used as positive data for ANN training** *

| Mutation | Gene | ERV family | Orientation | Distance to exon (bp) | Intron Size |
| --- | --- | --- | --- | --- | --- |
| *Ap3d1^mh2J^* | *Ap3d1* | IAP | + | 5 | 97 |
| *Atrn^mg^* | *Atrn* | IAP | - | 136 | 2249 |
| *Atrn^mg-L^* | *Atrn* | IAP | + | 420 | 1953 |
| *Eya1^bor^* | *Eya1* | IAP | + | 1575 | 20875 |
| *Gus^mps2J^* | *Gusb* | IAP | + | 975 | 2248 |
| *Lama2^Pas^* | *Lama2* | IAP | + | 300 | 9389 |
| *LamB3^IAP^* | *LamB3* | IAP | - | 1 | 1346 |
| *Mgrn1^md-2J^* | *Mgrn1* | IAP | + | 601 | 3233 |
| *Mgrn1^md^* | *Mgrn1* | IAP | + | 942 | 3142 |
| *Pitpna^vb^* | *Pitpna* | IAP | + | 1126 | 5624 |
| *Spna1^Dem^* | *Spna1* | IAP | + | 1 | 2796 |
| *Pofut1^cax^* | *Pofut1* | IAP | - | 24 | 9806 |
| *Pmca2^joggle^* | *Atp2b2* | IAP | + | 15 | 24786 |
| *Gria4^spkw1^* | *Gria4* | IAP | + | 720 | 4003 |
| *Zfp69^SJL^* | *Zfp69* | IAP | + | 965 | 12095 |
| *Adcy1^brl^* | *Adcy1* | ETn | + | 1700 | 9302 |
| *Cacng2^stg^* | *Cacng2* | ETn | + | 1800 | 16321 |
| *Cacng2^stg-3J^* | *Cacng2* | ETn | + | 3300 | 16321 |
| *Clcn1^adr^* | *Clcn1* | ETn | + | 1033 | 4868 |
| *Fas^lpr^* | *Fas* | ETn | + | 14 | 1757 |
| *Fbxw4^Dac-2J^* | *Fbxw4* | ETn | + | 13000 | 39070 |
| *Gli3^pdn^* | *Gli3* | ETn | + | 24514 | 65136 |
| *Hk1^dea^* | *Hk1* | ETn | + | 901 | 5450 |
| *Lep^0b-2J^* | *Lep* | ETn | + | 3200 | 8672 |
| *Mip^Cat-Fr^* | *Mip* | ETn | + | 800 | 2605 |
| *Muted^mu^* | *Muted* | ETn | + | 2362 | 6388 |
| *Ttc7^fsn^* | *Ttc7* | ETn | + | 57 | 4401 |
| *Hsf4^lop11^* | *Hsf4* | ETn | + | 61 | 1712 |
| *Fig4^paletremor^* | *Fig4* | ETn | + | 384 | 2016 |
| *Dysf ^prmd^* | *Dysf* | ETn | + | 495 | 4796 |
| *Zhx2^Afr1^* | *Zhx2* | ETn | + | 20600 | 67641 |
| *Slc6a5^mlJ^* | *Slc6a5* | MusD | + | 1833 | 9798 |
| *Rubie^Ecl^* | *Rubie* | ETn | + | 611 | 4429 |

* For references of the above cases, please refer to *Table 3* in *Text S2* of reference [11] in the main text.
