# Supplementary material for: Visualized Computational Predictions of Transcriptional Effects by Intronic Endogenous Retroviruses
Source: PLoS One. 2013 Aug 6;8(8):e71971. doi: 10.1371/journal.pone.0071971 (PMC3735543; doi:10.1371/journal.pone.0071971)
Supplement: Table S2 — Mouse common ERV insertions used as negative data for ANN training. (DOCX) [file pone.0071971.s004.docx]

**Table S2. Mouse common ERV insertions used as negative data for ANN training**

| Gene | ERV Family | Orientation | Distance to exon (bp) | Intron Size | Chromosome | Insertion* Start Site | Insertion End Site |
| --- | --- | --- | --- | --- | --- | --- | --- |
| Xkr4 | ETn | - | 57181 | 198446 | chr1 | 3264230 | 3270517 |
| A830018L16Rik | ETn | - | 23037 | 146107 | chr1 | 11609435 | 11614975 |
| Gm106 | ETn | - | 2022 | 17789 | chr1 | 15852454 | 15857959 |
| Ugt1a6b | ETn | - | 5188 | 100672 | chr1 | 90009562 | 90016134 |
| Nmnat2 | ETn | - | 945 | 3901 | chr1 | 154925062 | 154930510 |
| Gphn | ETn | - | 675 | 30387 | chr12 | 79507116 | 79512652 |
| Mctp1 | ETn | - | 64848 | 252665 | chr13 | 76587547 | 76593155 |
| Abcc4 | ETn | - | 4860 | 16033 | chr14 | 119042582 | 119048511 |
| Sntb1 | ETn | - | 29017 | 108236 | chr15 | 55703024 | 55708561 |
| Tbc1d12 | ETn | - | 3935 | 11602 | chr19 | 38944164 | 38949481 |
| Gm410 | ETn | - | 2606 | 11815 | chr3 | 57486131 | 57491629 |
| BC060267 | ETn | - | 575 | 8566 | chr6 | 115774304 | 115779810 |
| Clec2g | ETn | - | 1785 | 8846 | chr6 | 128886285 | 128891652 |
| Gvin1 | ETn | - | 8523 | 33780 | chr7 | 113344731 | 113350215 |
| Mtmr7 | ETn | - | 2628 | 11840 | chr8 | 41652051 | 41657555 |
| Gm6531 | ETn | - | 4413 | 11489 | chr8 | 87682996 | 87690809 |
| 1700008F21Rik | ETn | - | 6920 | 31685 | chr8 | 131621381 | 131626879 |
| Aox3l1 | IAP | - | 298 | 771 | chr1 | 58380192 | 58387315 |
| Casp8 | IAP | - | 5055 | 21005 | chr1 | 58868532 | 58875703 |
| Pard3b | IAP | - | 1263 | 47704 | chr1 | 62580617 | 62587869 |
| Map3k5 | IAP | - | 1769 | 11696 | chr10 | 19730438 | 19737594 |
| Slc2a12 | IAP | - | 1589 | 19104 | chr10 | 22403027 | 22410351 |
| Trpm2 | IAP | - | 1368 | 3353 | chr10 | 77413477 | 77420631 |
| Vrk2 | IAP | - | 5371 | 36172 | chr11 | 26455446 | 26460785 |
| Rapgef6 | IAP | - | 8084 | 35172 | chr11 | 54390082 | 54397201 |
| Usp32 | IAP | - | 2189 | 11582 | chr11 | 84874950 | 84881373 |
| Immp2l | IAP | + | 252601 | 885136 | chr12 | 42090131 | 42097215 |
| Rad51l1 | IAP | - | 88845 | 323296 | chr12 | 80662842 | 80669965 |
| Dcaf5 | IAP | - | 11771 | 24336 | chr12 | 81518250 | 81525400 |
| Adarb2 | IAP | - | 84261 | 350575 | chr13 | 8468880 | 8474088 |
| Amph | IAP | - | 44873 | 90242 | chr13 | 19085739 | 19092948 |
| F13a1 | IAP | - | 3507 | 14371 | chr13 | 37128592 | 37135718 |
| Sirt5 | IAP | + | 859 | 2040 | chr13 | 43482163 | 43489246 |
| Mctp1 | IAP | - | 110661 | 251121 | chr13 | 76633360 | 76640512 |
| Rgnef | IAP | - | 5396 | 60746 | chr13 | 98852671 | 98860156 |
| Rgs7bp | IAP | + | 8224 | 64348 | chr13 | 105781300 | 105786913 |
| Fhit | IAP | - | 27865 | 183134 | chr14 | 10561294 | 10568388 |
| Fhit | IAP | + | 235632 | 544223 | chr14 | 11011280 | 11018376 |
| Ube2e2 | IAP | + | 4887 | 233982 | chr14 | 19692040 | 19699120 |
| Adk | IAP | + | 3151 | 45346 | chr14 | 22179600 | 22185141 |
| Nrg3 | IAP | - | 47237 | 95126 | chr14 | 39268291 | 39275378 |
| Klhl1 | IAP | - | 3710 | 59482 | chr14 | 96683144 | 96690216 |
| Gpc6 | IAP | + | 1087 | 253968 | chr14 | 117578198 | 117584796 |
| Gpc6 | IAP | - | 98934 | 241714 | chr14 | 117684976 | 117692093 |
| Itgbl1 | IAP | + | 62953 | 160414 | chr14 | 124123683 | 124129512 |
| Wdr70 | IAP | - | 2483 | 24185 | chr15 | 7860878 | 7868003 |
| Itgb5 | IAP | - | 2167 | 15025 | chr16 | 33847324 | 33852675 |
| Hlcs | IAP | - | 5831 | 12039 | chr16 | 94496513 | 94503567 |
| Park2 | IAP | - | 30559 | 163009 | chr17 | 11290717 | 11297877 |
| Wdr27 | IAP | - | 4555 | 39775 | chr17 | 15001807 | 15008545 |
| H2-T24 | IAP | - | 313 | 668 | chr17 | 36144089 | 36151188 |
| Capn11 | IAP | - | 775 | 4036 | chr17 | 45781596 | 45786870 |
| Tmem232 | IAP | - | 7764 | 19085 | chr17 | 65811104 | 65818193 |
| Alk | IAP | - | 69170 | 215491 | chr17 | 72798671 | 72806046 |
| Nrxn1 | IAP | - | 42869 | 95437 | chr17 | 90501772 | 90508885 |
| Mpp7 | IAP | - | 14524 | 41586 | chr18 | 7527584 | 7534661 |
| Fam59a | IAP | - | 25630 | 56393 | chr18 | 21420272 | 21427387 |
| Pcdha7 | IAP | - | 5252 | 172754 | chr18 | 37141184 | 37148306 |
| Dctn4 | IAP | - | 869 | 2872 | chr18 | 60688022 | 60693377 |
| Dok6 | IAP | - | 17028 | 165225 | chr18 | 89785211 | 89790265 |
| Ankrd22 | IAP | - | 2297 | 13254 | chr19 | 34205644 | 34212748 |
| Tll2 | IAP | - | 2552 | 12199 | chr19 | 41217306 | 41224389 |
| Nebl | IAP | - | 22365 | 133805 | chr2 | 17534169 | 17539555 |
| Shc4 | IAP | - | 1060 | 4454 | chr2 | 125456566 | 125461605 |
| Macrod2 | IAP | - | 5836 | 26305 | chr2 | 142022880 | 142029999 |
| Macrod2 | IAP | - | 6390 | 15828 | chr2 | 142131770 | 142137226 |
| Pag1 | IAP | - | 14088 | 38967 | chr3 | 9731168 | 9738324 |
| Ralyl | IAP | - | 44161 | 257337 | chr3 | 13821264 | 13826537 |
| Wdr63 | IAP | - | 512 | 2982 | chr3 | 145746551 | 145753362 |
| Gm3893 | IAP | - | 11131 | 138699 | chr4 | 41971075 | 41976105 |
| Cyp2j13 | IAP | - | 758 | 3869 | chr4 | 95715430 | 95722490 |
| Inadl | IAP | - | 1367 | 4919 | chr4 | 98191559 | 98198648 |
| Zyg11b | IAP | - | 9736 | 21427 | chr4 | 107956679 | 107963759 |
| Nipal3 | IAP | - | 907 | 4139 | chr4 | 135011719 | 135018793 |
| Gm13152 | IAP | + | 26677 | 327971 | chr4 | 146576690 | 146583567 |
| Gm13152 | IAP | - | 37470 | 327534 | chr4 | 146840077 | 146847391 |
| Lphn3 | IAP | + | 2971 | 50711 | chr5 | 81806725 | 81813828 |
| Slc4a4 | IAP | - | 20317 | 66270 | chr5 | 89429935 | 89437000 |
| Scarb2 | IAP | - | 3097 | 17119 | chr5 | 92905132 | 92911125 |
| Ica1 | IAP | - | 23732 | 57499 | chr6 | 8646155 | 8653249 |
| Exoc4 | IAP | + | 8780 | 172353 | chr6 | 33693651 | 33699186 |
| Grid2 | IAP | - | 60057 | 174231 | chr6 | 64104490 | 64111614 |
| C130060K24Rik | IAP | - | 3197 | 52475 | chr6 | 65334821 | 65341902 |
| Frmd4b | IAP | - | 16742 | 34938 | chr6 | 97322251 | 97329444 |
| Slco1a6 | IAP | - | 1170 | 4372 | chr6 | 142097493 | 142104585 |
| Far2 | IAP | - | 605 | 2529 | chr6 | 148114872 | 148119995 |
| 2900092C05Rik | IAP | - | 2556 | 29668 | chr7 | 13127958 | 13133276 |
| Mrgprx2 | IAP | + | 4151 | 10585 | chr7 | 55742578 | 55748086 |
| Tcerg1l | IAP | - | 1796 | 11330 | chr7 | 145453431 | 145458734 |
| Tubgcp2 | IAP | - | 6665 | 15153 | chr7 | 147200658 | 147207237 |
| Shank2 | IAP | - | 40865 | 103661 | chr7 | 151535467 | 151540502 |
| Adam5 | IAP | - | 6968 | 26813 | chr8 | 25877885 | 25885069 |
| Kcnu1 | IAP | - | 2755 | 8510 | chr8 | 26981928 | 26989013 |
| Enpp6 | IAP | - | 1525 | 5212 | chr8 | 48169417 | 48174660 |
| Nek1 | IAP | - | 564 | 5772 | chr8 | 63477768 | 63484861 |
| March1 | IAP | - | 36422 | 81716 | chr8 | 68448599 | 68454523 |
| Psd3 | IAP | - | 1771 | 84538 | chr8 | 70316611 | 70324112 |
| Large | IAP | - | 13646 | 130313 | chr8 | 75449705 | 75455525 |
| Nr3c2 | IAP | - | 11964 | 63197 | chr8 | 79660798 | 79667136 |
| Vat1l | IAP | - | 34721 | 74172 | chr8 | 116848063 | 116854300 |
| Wwox | IAP | + | 63882 | 633554 | chr8 | 117805823 | 117811655 |
| 2310061C15Rik | IAP | - | 2107 | 9807 | chr8 | 119420166 | 119427327 |
| Pkd1l2 | IAP | + | 1854 | 4436 | chr8 | 119526465 | 119531615 |
| Cdh13 | IAP | + | 42827 | 119831 | chr8 | 121569097 | 121576257 |
| Cwf19l2 | IAP | - | 1163 | 10172 | chr9 | 3461294 | 3466473 |
| Gria4 | IAP | - | 3914 | 14912 | chr9 | 4491286 | 4498459 |
| Opcml | IAP | + | 183045 | 605762 | chr9 | 27782076 | 27789195 |
| Ccdc15 | IAP | - | 3958 | 14817 | chr9 | 37138954 | 37146062 |
| Larp6 | IAP | - | 1859 | 5539 | chr9 | 60574023 | 60581116 |
| Slc17a5 | IAP | + | 2071 | 5088 | chr9 | 78410684 | 78415811 |
| Mei4 | IAP | - | 14167 | 30248 | chr9 | 81800055 | 81806537 |
| Lrrc2 | IAP | - | 290 | 3892 | chr9 | 110872953 | 110878371 |
| Cdcp1 | IAP | + | 702 | 18321 | chr9 | 123099950 | 123107326 |
| Mageb18 | IAP | - | 123284 | 390950 | chrX | 89493715 | 89500415 |
| Il1rapl2 | IAP | - | 37549 | 515610 | chrX | 134223889 | 134231006 |
| Ctps2 | IAP | - | 3354 | 14300 | chrX | 159416565 | 159423690 |
| Frmpd4 | IAP | - | 266328 | 545303 | chrX | 164614545 | 164619551 |

*Genomic coordinates are from the mm9 version of the reference mouse genome.
