# Supplementary material for: Visualized Computational Predictions of Transcriptional Effects by Intronic Endogenous Retroviruses
Source: PLoS One. 2013 Aug 6;8(8):e71971. doi: 10.1371/journal.pone.0071971 (PMC3735543; doi:10.1371/journal.pone.0071971)
Supplement: Table S3 — Mouse polymorphic ERV insertions with predicted likelihood of affecting gene transcription. (DOCX) [file pone.0071971.s005.docx]

**Table S3. Mouse polymorphic ERV insertions with predicted likelihood of affecting gene transcription**

| Gene | ERV Family | Orientation | Distance to exon | Intron Size | Prediction | Known as Positive |
| --- | --- | --- | --- | --- | --- | --- |
| Nsdhl | IAP | + | 57 | 12228 | 0.987909781 |  |
| Adamts13 | IAP | + | 425 | 1809 | 0.961451222 | Yes [1] |
| Trpc6 | IAP | + | 439 | 1934 | 0.958190312 | Yes [2] |
| Mnd1 | IAP | + | 618 | 6673 | 0.870455213 |  |
| 4930401O12Rik | IAP | + | 512 | 11783 | 0.869037367 |  |
| Slc20a2 | IAP | + | 647 | 6215 | 0.865231215 |  |
| Poteg | IAP | + | 830 | 2660 | 0.851299045 |  |
| Dph5 | IAP | + | 598 | 9923 | 0.84938053 |  |
| Zfp69 | IAP | + | 964 | 4990 | 0.769065253 | Yes [3] |
| Dnahc6 | IAP | - | 40 | 1793 | 0.562173545 |  |
| Cdk5rap1 | IAP | - | 53 | 1016 | 0.544559713 | Yes [4] |
| Slc15a2 | IAP | + | 1710 | 4378 | 0.354473968 | Yes [5] |
| 2010111I01Rik | IAP | - | 159 | 7374 | 0.344009385 |  |
| Nudcd3 | IAP | + | 1863 | 17814 | 0.297287233 |  |
| Pion | IAP | - | 201 | 6373 | 0.24039571 |  |
| Vnn3 | ETn | - | 310 | 2251 | 0.217773905 |  |
| Gm6034 | IAP | - | 225 | 6028 | 0.176489314 |  |
| Kcnh6 | IAP | - | 221 | 730 | 0.152783831 | Yes [2] |
| Rgs3 | IAP | + | 2809 | 9751 | 0.134024115 |  |
| Ccrn4l | IAP | + | 2502 | 17183 | 0.124475592 | Yes [6,7] |
| Tbc1d22a | IAP | - | 423 | 34482 | 0.06374926 |  |
| Zfp933 | IAP | + | 4706 | 12324 | 0.062201808 |  |
| Agtr1a | IAP | + | 3666 | 28774 | 0.058699153 |  |
| Gm14459 | IAP | - | 301 | 615 | 0.050337372 |  |
| Me3 | IAP | + | 7702 | 19746 | 0.037886502 |  |
| Kcns3 | IAP | + | 9019 | 19924 | 0.03494976 |  |
| 4930565D16Rik | IAP | + | 9055 | 20880 | 0.034365384 |  |
| Scg5 | IAP | + | 10331 | 28244 | 0.029424707 |  |
| Gbe1 | IAP | + | 13228 | 26664 | 0.027582524 |  |
| L3mbtl4 | IAP | + | 10436 | 33214 | 0.027430072 |  |
| Agbl4 | IAP | + | 5020 | 42822 | 0.027124412 |  |
| Lrrc33 | IAP | - | 404 | 9301 | 0.024461052 |  |
| Pla2g4e | IAP | + | 17216 | 37311 | 0.021845047 |  |
| Sgsm2 | IAP | - | 491 | 15725 | 0.018014046 |  |
| Snx29 | IAP | + | 19387 | 48991 | 0.017131728 |  |
| Tmem163 | IAP | + | 5454 | 108619 | 0.015825218 |  |
| Ccdc46 | IAP | + | 9256 | 52761 | 0.012623208 |  |
| Wiz | ETn | - | 1275 | 5841 | 0.012248221 | Yes [8] |
| 4930558C23Rik | ETn | - | 1158 | 10137 | 0.011952727 |  |
| Gdpd4 | IAP | - | 526 | 1509 | 0.007326896 |  |
| Mir4660 | IAP | - | 1096 | 100521 | 0.006968322 |  |
| Polr1a | IAP | - | 582 | 1344 | 0.005842897 | Yes [5] |
| Sesn1 | IAP | + | 29232 | 75637 | 0.005304333 |  |
| Tek | IAP | - | 570 | 2397 | 0.005265554 |  |
| Trim14 | IAP | - | 558 | 4899 | 0.005249294 |  |
| Psmg4 | IAP | - | 638 | 4613 | 0.003616963 |  |
| Csmd1 | IAP | + | 79475 | 311125 | 0.002736655 |  |
| Schip1 | IAP | + | 49450 | 424426 | 0.002723056 |  |
| Pde4d | IAP | + | 44723 | 123735 | 0.002676208 |  |
| Slc4a4 | ETn | - | 3961 | 9103 | 0.002644464 |  |
| Fmo1 | IAP | - | 778 | 3196 | 0.002415332 |  |
| Ttbk2 | ETn | - | 4957 | 10925 | 0.002093846 |  |
| Cd84 | ETn | - | 5024 | 13320 | 0.001979836 |  |
| Sh3bp4 | ETn | - | 4942 | 31110 | 0.001689605 |  |
| Atp6v1e1 | IAP | - | 946 | 4737 | 0.001568058 |  |
| Rspry1 | IAP | - | 960 | 15042 | 0.001550676 |  |
| Gpsm1 | IAP | - | 1161 | 6967 | 0.001086912 |  |
| Pld1 | IAP | - | 1194 | 7391 | 0.001039198 |  |
| BC117090 | IAP | - | 1338 | 4076 | 0.000965293 |  |
| Bst1 | IAP | - | 1372 | 3845 | 0.000944359 |  |
| Zfp277 | IAP | - | 1373 | 24591 | 0.000873791 |  |
| Gm4788 | IAP | - | 1403 | 24191 | 0.000842515 |  |
| Agl | IAP | - | 1499 | 4800 | 0.000826516 |  |
| Ttbk2 | IAP | - | 1427 | 8446 | 0.000819854 |  |
| Ccdc158 | IAP | - | 1594 | 3535 | 0.000806905 |  |
| Rnf157 | IAP | - | 1478 | 28510 | 0.000801104 |  |
| Nipa2 | IAP | - | 1683 | 9180 | 0.000684396 |  |
| Kalrn | IAP | - | 2033 | 4143 | 0.000629023 |  |
| Raver2 | IAP | - | 2042 | 5517 | 0.000607147 |  |
| Nell1 | IAP | - | 2094 | 12175 | 0.000555182 |  |
| Mc2r | IAP | - | 2326 | 5044 | 0.000554855 |  |
| Lhfp | ETn | - | 69728 | 182558 | 0.000506862 |  |
| Cntnap3 | IAP | - | 2312 | 21581 | 0.000500788 |  |
| Plcl2 | IAP | - | 2355 | 22759 | 0.000493444 |  |
| Mier3 | IAP | - | 2805 | 5659 | 0.000485286 |  |
| Lrrc8d | IAP | - | 2833 | 25902 | 0.00043732 |  |
| Pet112 | IAP | - | 3269 | 10800 | 0.000425189 |  |
| Smad5 | IAP | - | 3450 | 12519 | 0.000410445 |  |
| Lrrc8b | IAP | - | 3424 | 47513 | 0.000389703 |  |
| Ttc27 | IAP | - | 3963 | 11906 | 0.000387058 |  |
| Hsf3 | IAP | - | 4120 | 13387 | 0.000378471 |  |
| Slc2a9 | IAP | - | 4324 | 11126 | 0.000375052 |  |
| Slc24a3 | IAP | - | 4737 | 12030 | 0.00036089 |  |
| Kif24 | IAP | - | 4708 | 30461 | 0.000346866 |  |
| Sgip1 | IAP | - | 4938 | 22628 | 0.00034535 |  |
| Abhd2 | IAP | - | 5662 | 17127 | 0.000334478 |  |
| Arfgef2 | IAP | - | 6364 | 15820 | 0.000324289 |  |
| Mmrn2 | IAP | - | 7020 | 15097 | 0.000316362 |  |
| 4930405D11Rik | IAP | - | 6328 | 66088 | 0.000309559 |  |
| Enpp1 | IAP | - | 7171 | 27361 | 0.000307803 |  |
| 2010111I01Rik | IAP | - | 7835 | 24773 | 0.000302316 |  |
| Vegfc | IAP | - | 6921 | 71884 | 0.000301886 |  |
| Cacna2d1 | IAP | - | 7946 | 25383 | 0.000301083 |  |
| Exoc6 | IAP | - | 8331 | 23466 | 0.000298629 |  |
| 2210408I21Rik | IAP | - | 7207 | 271055 | 0.000296854 |  |
| Gpc6 | IAP | - | 7253 | 238881 | 0.000296398 |  |
| E330023G01Rik | IAP | - | 8128 | 56365 | 0.000292563 |  |
| Sdk2 | IAP | - | 9044 | 32860 | 0.000290307 |  |
| Osbpl10 | IAP | - | 10702 | 34893 | 0.000280429 |  |
| Gm20752 | IAP | - | 11152 | 44522 | 0.000276596 |  |
| Cdh18 | IAP | - | 12143 | 25549 | 0.000276526 |  |
| 4930544M13Rik | IAP | - | 10708 | 66199 | 0.000275969 |  |
| Cntnap5a | IAP | - | 10224 | 210264 | 0.000275844 |  |
| 4932441J04Rik | IAP | - | 13110 | 42474 | 0.000269509 |  |
| Pik3c2g | IAP | - | 12510 | 66056 | 0.000268826 |  |
| Trak1 | IAP | - | 14358 | 32329 | 0.00026747 |  |
| Lrp1b | IAP | - | 15560 | 34950 | 0.000263811 |  |
| 4933400C23Rik | IAP | - | 15588 | 46932 | 0.00026201 |  |
| Mast4 | IAP | - | 16535 | 36106 | 0.000261362 |  |
| Tmem132b | IAP | - | 16632 | 43391 | 0.000260101 |  |
| Ppm1h | IAP | - | 17145 | 38451 | 0.00025971 |  |
| Anks1b | IAP | - | 16924 | 46226 | 0.000259141 |  |
| Mbnl1 | IAP | - | 16700 | 117208 | 0.000255769 |  |
| Nckap5 | IAP | - | 18758 | 60598 | 0.000254342 |  |
| Gfod1 | IAP | - | 18587 | 94886 | 0.000252576 |  |
| Reln | IAP | - | 21091 | 50847 | 0.000251614 |  |
| Rap1gap2 | IAP | - | 21818 | 77024 | 0.000248785 |  |
| Camk1d | IAP | - | 23016 | 141262 | 0.000246287 |  |
| Atrnl1 | IAP | - | 29606 | 132697 | 0.000240227 |  |
| Gabrb1 | IAP | - | 31538 | 71302 | 0.000240039 |  |
| Akap6 | IAP | - | 30663 | 91189 | 0.000239778 |  |
| Tenm4 | IAP | - | 30646 | 137484 | 0.000239474 |  |
| Bank1 | IAP | - | 32945 | 102796 | 0.000237951 |  |
| Ninj2 | IAP | - | 34146 | 97379 | 0.00023731 |  |
| Fras1 | IAP | - | 37811 | 137210 | 0.000235242 |  |
| Gm5095 | IAP | - | 48980 | 158923 | 0.00023075 |  |
| Pdzd2 | IAP | - | 60260 | 128735 | 0.000230422 |  |
| Negr1 | IAP | - | 54674 | 291865 | 0.000230422 |  |
| Luzp2 | IAP | - | 102994 | 209785 | 0.000230422 |  |
| Dlg2 | IAP | - | 54093 | 152758 | 0.000230422 |  |
| Dip2c | IAP | - | 53209 | 208802 | 0.000230422 |  |
| Csmd1 | IAP | - | 123758 | 309417 | 0.000230422 |  |
| Cntnap2 | IAP | - | 74080 | 268475 | 0.000230422 |  |
| Accn1 | IAP | - | 414937 | 988804 | 0.000230422 |  |

**References**

1. Banno F, Kaminaka K, Soejima K, Kokame K, Miyata T (2004) Identification of strain-specific variants of mouse Adamts13 gene encoding von Willebrand factor-cleaving protease. The Journal of biological chemistry 279: 30896–30903. doi:10.1074/jbc.M314184200.

2. Zhang Y, Romanish MT, Mager DL (2011) Distributions of transposable elements reveal hazardous zones in Mammalian introns. PLoS Comput Biol 7: e1002046. doi:10.1371/journal.pcbi.1002046.

3. Scherneck S, Nestler M, Vogel H, Blüher M, Block M-D, et al. (2009) Positional Cloning of Zinc Finger Domain Transcription Factor Zfp69, a Candidate Gene for Obesity-Associated Diabetes Contributed by Mouse Locus Nidd/SJL. PLoS genetics 5: e1000541. doi:10.1371/journal.pgen.1000541.

4. Druker R, Bruxner TJ, Lehrbach NJ, Whitelaw E (2004) Complex patterns of transcription at the insertion site of a retrotransposon in the mouse. Nucleic acids research 32: 5800–5808. doi:10.1093/nar/gkh914.

5. Li J, Akagi K, Hu Y, Trivett AL, Hlynialuk CJ, et al. (2012) Mouse endogenous retroviruses can trigger premature transcriptional termination at a distance. Genome research. doi:10.1101/gr.130740.111.

6. Barbot W, Dupressoir A, Lazar V, Heidmann T (2002) Epigenetic regulation of an IAP retrotransposon in the aging mouse: progressive demethylation and de-silencing of the element by its repetitive induction. Nucleic acids research 30: 2365–2373.

7. Puech A, Dupressoir A, Loireau MP, Mattei MG, Heidmann T (1997) Characterization of two age-induced intracisternal A-particle-related transcripts in the mouse liver. Transcriptional read-through into an open reading frame with similarities to the yeast ccr4 transcription factor. The Journal of biological chemistry 272: 5995–6003.

8. Baust C, Baillie GJ, Mager DL (2002) Insertional polymorphisms of ETn retrotransposons include a disruption of the wiz gene in C57BL/6 mice. Mammalian genome : official journal of the International Mammalian Genome Society 13: 423–428. doi:10.1007/s00335-002-2178-3.
